# Taking a Step Further in Identifying Ideal Blood Pressure Range Among Hemodialysis Patients: A Systematic Review and a Meta-Analysis

**DOI:** 10.3389/fphar.2020.00729

**Published:** 2020-05-26

**Authors:** Raja Ahsan Aftab, Renukha Sellappans, Cheong Kah Ming, Imam Shaik

**Affiliations:** ^1^Faculty of Health and Medical Sciences, School of Pharmacy, Taylor's University, Subang Jaya, Malaysia; ^2^Faculty of Health and Medical Science, School of Medicine, Taylor's University, Subang Jaya, Malaysia

**Keywords:** blood pressure, hypertension, end-stage renal disease, mortality, survival

## Abstract

**Background:**

Hypertension is one of the primary predictor of mortality among end-stage renal disease (ESRD) patients on dialysis. However, there is no consensus on an ideal blood pressure range for this population.

**Aims and Objective:**

To identify an ideal systolic blood pressure range based on optimal survival among ESRD patients on dialysis.

**Method:**

A systematic search for clinical trials assessing the impact of different systolic blood pressure range on mortality among ESRD patients on hemodialysis was conducted through PubMed, EBSCOhost, Science Direct, Google Scholar, and Scopus. All randomized control trials (RCTs) involving ESRD patients on hemodialysis with primary or secondary outcome of assessing the impact different systolic blood pressure range (< 140 and >140 mm Hg) on all-cause mortality were included. The quality of reporting of the included studies was evaluated using the Jadad scale. Two researchers independently conducted eligibility assessment. Discrepancies were resolved by discussion and consultation with a third researcher when needed. Pooled relative risks (RRs) with 95% confidence intervals (CIs) were calculated.

**Results:**

A total of 1,787 research articles were identified during the initial search, after which six RCTs met our inclusion criteria. According to the Jadad scale, all six RCTs scored 3 points each for quality of reporting. Four RCTs employed pharmacological intervention while two RCTs assessed non-pharmacological intervention. Of the six RCTs, two studies were able to achieve a systolic blood pressure of <140 mm Hg at the end of trial with a RR for reduction in mortality of 0.56 (95% CI, 0.3–1.07; *P* = 0.08). Four RCTs were able to achieve a systolic blood pressure of >140 mm Hg at the end of trial, with the RR for reduction of mortality of 0.72 (95% CI, 0.54–0.96; *P* = 0.003). Overall, pooled estimates of the six RCTs suggested the reduction in systolic blood pressure statistically reduce all cause of mortality (RR, 0.69%; 95% CI, 0.53–0.90; *P* = 0.006) among ESRD patients on hemodialysis.

**Conclusion:**

Though not statically significant, the current study identifies <140 mm Hg as a promising blood pressure range for optimum survival among ESRD patients on hemodialysis. However, further studies are required to establish an ideal blood pressure range among hemodialysis patients.

**Systematic Review Registration:**

The study protocol was registered under PROSPERO (CRD42019121102).

## Introduction

Hypertension among end-stage renal disease (ESRD) patients on hemodialysis is common and is one of the main contributors towards high mortality and morbidity among these patients. Several studies have shown a “U-shape” relationship between high blood pressure and mortality, implying that blood pressure levels below certain range is more harmful than higher levels (Carney, 2013). Hypertension being multifactorial in nature and this reverse epidemiology of blood pressure and mortality makes it difficult to determine blood pressure targets especially in high-risk patients such as ESRD patients on hemodialysis (Taniyama, 2016).

Benefits of lowering blood pressure in hypertensive patients have long been established through randomized controlled trials (RCTs). The SPRINT trial reported the beneficial effects of reducing blood pressure to 120 mm Hg, which include reduction in fatal and non-fatal cardiovascular events and overall mortality rates (Wright et al., 2015). However, high-risk individuals were excluded from the trial including those with diabetes and cerebrovascular disease, which limits its generalizability to this population. Furthermore, evidence of protective effects of pharmacologically-induced blood pressure reductions in patients with comorbidities remains uncertain (Patel et al., 2007; Wright et al., 2015). Thereby, optimal blood pressure targets especially among hemodialysis patients remains debatable.

Given the methods and timing of blood pressure measurements are variable, the relationship between hypertension and mortality among hemodialysis patients is complex. Clinical practice guidelines suggest a pre-dialysis blood pressure of <140/90 mm Hg and post dialysis blood pressure of <130/80 mm Hg (K/DOQI Workgroup, 2005). However, the evidence to support these recommendations are weak as it was extrapolated from observational studies or data derived from non-ESRD patients (Chang, 2011). On the other hand, studies have reported that a high percentage of patients achieving these targeted blood pressure range suffered from intradialytic hypotension, which may lead to myocardial stunning phenomena and death (McIntyre and Goldsmith, 2015; MacEwen et al., 2017; McCallum and Sarnak, 2019).

This ambiguity and uncertainty revolving around ideal blood pressure ranges among hemodialysis patients prompted the authors to perform a systematic review and meta-analysis to identify ideal systolic blood pressure that corresponds to optimal survival among hemodialysis patients.

## Method

The preferred reporting framework for systematic review and meta-analysis, the PRISMA statement was strictly adhered to while performing the literature search and reporting of results.

### Data Sources and Search Strategy

A systematic search for clinical trials assessing the impact of different systolic blood pressure ranges (<140 and >140 mm Hg) on mortality among ESRD patients on hemodialysis was conducted. PubMed, EBSCO host, Science Direct, Google Scholar, and Scopus search engines were searched for original research articles published to date (March 2019).

The keywords and MESH terms used were *hypertension, blood pressure, dialysis, end-stage renal disease, end stage renal failure, mortality, survival*, and *death*. We limited the search results to clinical trials and articles published in English.

### Study Selection

The inclusion criteria applied were RCTs with primary or secondary outcome of assessing the impact different systolic blood pressure range (<140 and >140 mm Hg) on all-cause mortality among ESRD, hypertensive adults (>18 years old) on regular hemodialysis for at least 1 year. Both pharmacological and non-pharmacological intervention that was administered for at least 6 months to reduce blood pressure were included. Only studies with a minimum follow-up period of 1 year and published in English language were included.

Two researchers independently reviewed the titles and abstracts of all potential research articles that met the inclusion and exclusion criteria. Any differences were resolved through discussion or by consulting a third reviewer. Full texts of the eligible research articles were retrieved and screened for eligibility by two researchers independently. Any differences were resolved through discussion or by a third reviewer.

### Quality of Reporting

The Jadad scale was used to assess the quality of reporting of RCT in this study. The Jadad scale is a three-item scale assessing the randomization, blinding, and drop-out methods. For each item, a study was awarded one point if it described randomization, double blind or dropout, respectively. If randomization or blinding method is judged appropriate, an additional one point was awarded for each item. Conversely, if the randomization or blinding method is judged inappropriate, one point was deducted from that item. Since randomization procedure is fundamental to the quality of RCT, one point was deducted if randomization procedure was not described. Blinding was considered appropriate if the article specifies whom the blinding involved. The original Jadad scale only considers double blinding as an appropriate method. Keeping in view of our objectives, we scored single blinding as an appropriate method as well. Next, articles were assessed with regards to information on patient withdrawal or exclusion after enrollment for any reason. These included decision by patient or investigator, loss to follow-up, change in treatment or any reason that may introduce bias. Articles were also assessed if they have mentioned or were clear from data shown that all patients were included in final outcome analysis or provided details of patient dropout. The final quality score for each article may range from 0 (lowest quality) to 5 (highest quality) (Burton et al., 2009).

### Data Extraction

Following review and selection of the full texts, one researcher independently extracted data from each included study and recorded them on a standardized data extraction sheet.

### Strategy for Data Synthesis

Quantitative synthesis or meta-analysis was performed using REVMAN 5 software. The number of all-cause mortality in each group and the relative risk (RR) with 95% confidence interval (CI) were calculated. Authors performed subgroup analysis based on systolic blood pressure >140 and <140 mm Hg and all-cause mortality reported in the included studies. Heterogeneity between studies were assessed using both the Chi-square test and the I^2^ statistic. A fixed effect model was used when I^2^ < 50%, which indicated heterogeneity. If I^2^ > 50%, a random effects model was used after consideration of the potential sources of heterogeneity. Publication bias was assessed using a funnel plot.

## Results

### Literature Search and Study Selection

**Figure 1:**
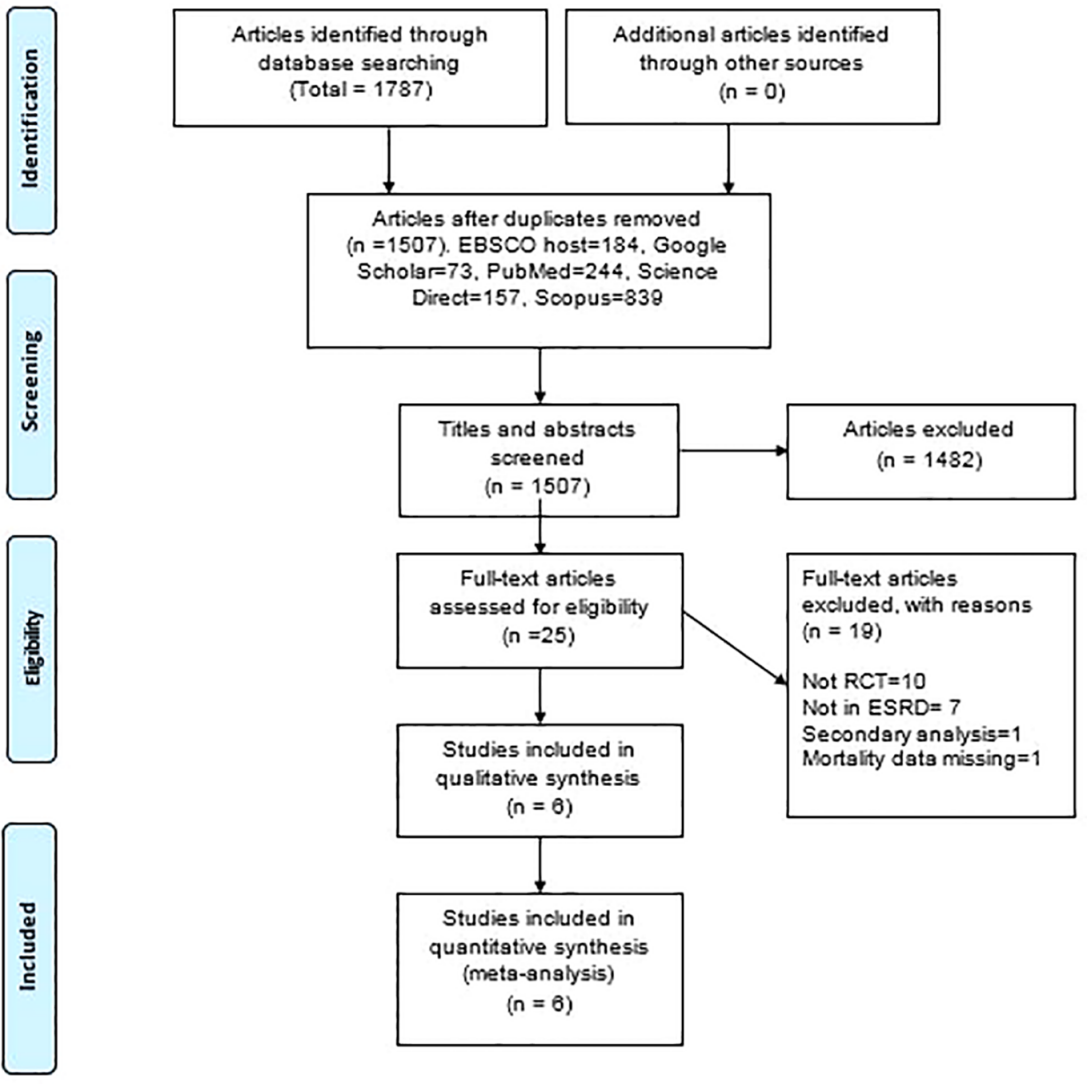
PRISMA flow diagram. RCT, randomized controlled trial; ESRD, end-stage renal disease.

A total of 1,787 articles were identified during the initial search. Following the elimination of 280 duplicate articles, 1,507 titles and abstracts were screened against the inclusion and exclusion criteria, and a total of 25 articles met the inclusion criteria (**Figure 1**). Out of this, 19 articles were excluded upon full text review as they were missing relevant data to meet the review objective. A final of six articles with a total of 1,306 patients (659 intervention arm and 647 standard arm) were selected for further analysis.

### Quality of Reporting

All six selected studies scored three points each according to the Jadad scale thereby suggesting that the quality of reporting was satisfactory. All six studies secured maximum points for describing the randomization process adequately along with withdrawal and dropouts. Three (50%) studies were not given any points for blinding domain as they did not include information on blinding (**Table 1**).

**Table 1:** Summary of quality of reporting for selected studies based on jaded scale.

Item	Points	Chertow et al., 2010	Santoro et al., 2008	Suzuki et al., 2008	Iseki et al., 2013	Takahashi et al., 2006	Aftab et al., 2017
Randomization							
Study Described as randomized	**+1**	+1	+1	+1	+1	+1	+1
Randomized method described and appropriate	**+1**	+1	+1	+1	+1	+1	+1
Randomized method described and inappropriate	−**1**	–	–	–	–	–	–
Randomization method not described	−**1**	–	–	–	–	–	–
**Blinding**							
Study described as double or single blind	**+1**	–	–	–	+1	+1	+1
Blinding method described and appropriate	**+1**	–	–	–	–	–	–
Blinding method described and inappropriate	−**1**	–	–	–	-1	-1	-1
Blinding method not described	**0**	–	–	–	–	–	–
Study not described as blinded	**0**	0	0	0	–	–	–
**Withdrawal and Dropouts**							
Withdrawals and dropouts described	**+1**	+1	+1	+1	+1	+1	+1
Withdrawals and dropouts not described	**0**	–	–	–	–	–	–
**Total**		**3**	**3**	**3**	**3**	**3**	**3**

### Study Characteristics

The six studies selected were published from 2006 to 2017. Of the six studies selected, four studies included pharmacological intervention whereas two studies assessed non-pharmacological intervention. All four studies that employed pharmacological intervention used angiotension receptor blocker in their trials. Studies by Chertow et al. (2010) and Santoro et al. (2008) used non-pharmacological intervention. These include high flux hemofilteration by Santoro et al. (2008) and frequent hemodialysis (six times/week) by Chertow et al. (2010). Follow up of patients ranged from 12 to 42 months while number of participants of individual study ranged from 64 Santoro et al. (2008) to 469 Iseki et al. (2013).

All six studies reported all-cause mortality as one of their outcomes. Altogether there were 188 all-cause mortality reported; 77 all-cause mortality were reported in the intervention arm while 111 all-cause of mortality were reported in the standard arm. Study by Takahashi et al. (2006), only reported mortality among standard arm patients however there was no mortality reported among intervention arm patients. Of the six studies, five (83.3%) reported a noticeable decline in blood pressure at the end of the study. However, Takahashi et al. (2006) reported that the decline in blood pressure did not significantly vary from baseline reading among the intervention arm patients. **Table 2** qualitatively summarizes the findings of the selected studies.

**Table 2:** Qualitative summary of selected studies.

Authors (Year)	Study type	Trial period	Standard treatment	Intervention treatment	All-cause mortality (n)	Baseline BP (mm Hg)	Post-trial BP (mm Hg)
Takahashi et al. (2006)	Prospective, randomized, open blinded-endpoint trial	3 years	Standard anti-hypertensive medication, n = 37	Candesartan, n = 43	Standard = 7 Intervention = 0	Control: 152 ± 4/85 ± 3 Intervention: 153 ± 2/82 ± 2	Control: 149 ± 3/80 ± 2 Intervention: 153 ± 2/83 ± 1
Suzuki et al. (2008)	Open-labeled randomized trial	3 years	Standard anti-hypertensive medication without ARBs, n = 180	ARBs (valsartan, candesartan, and losartan), n = 180	Standard = 38 Intervention = 25	Control: 145 ± 20/78 ± 12 Intervention: 141 ± 22/78 ± 14	Control: 140 ± 11/78 ± 7 Intervention: 140 ± 12/80 ± 8
Santoro et al. (2008)	Prospective, centrally randomized study with no blinding and based on the intention-to-treat principle	3 years	Ultrapure low-flux hemodialysis, n = 32	High-flux hemofiltration, n = 32	Standard = 12 Intervention = 7	Standard: 125.0 ± 3.4/72.0 ± 1.7 Intervention: 135.0 ± 4.3/78.0 ± 2.0	Standard: 112.1/67.6 Intervention: 127.6/75.9
Chertow et al. (2010)	Prospective, randomized, parallel-group trial	1 year	Conventional hemodialysis, three times/week, n = 120	Frequent hemodialysis, six times/week, n = 125	Standard = 9 Intervention = 5	Standard: 146 ± 18 Intervention: 147 ± 9	Standard: 147 ± 18 Intervention: 137 ± 19
Iseki et al. (2013)	Prospective, randomized, open-label, blinded-endpoint trial	3.5 years	Standard, non-ARB treatment, n = 234	(ARB) olmesartan, n = 235	Standard = 39 Intervention = 38	Standard: 160/81 Intervention: 159/80	Standard: 152.6/77.7 Intervention: 151.7/77.7
Aftab et al. (2017)	Prospective, randomized, parallel design, single-blind trial	1 year	Standard antihypertensive therapy, n = 44	Losartan (ARB), n = 44	Standard = 6 Intervention = 2	Standard: 157.5 ± 14.3 Intervention: 156.3 ± 13.4	Standard: 156.8 ± 11.3 Intervention: 149.7 ± 10.2

ARB, angiotensin receptor blocker; BP, blood pressure

### Systolic Blood Pressure <140 mm Hg and Mortality

Overall, two studies were able to achieve systolic blood pressure <140 mm Hg at the end of the trial, and both the studies employed non-pharmacological interventions. Altogether, there were 152 patients in control arm compared to 157 in the intervention arm. The total mortality among control arm patients was 21 (13.8%) compared to 12 (7.6%) in the intervention arm.

**Figure 2:**
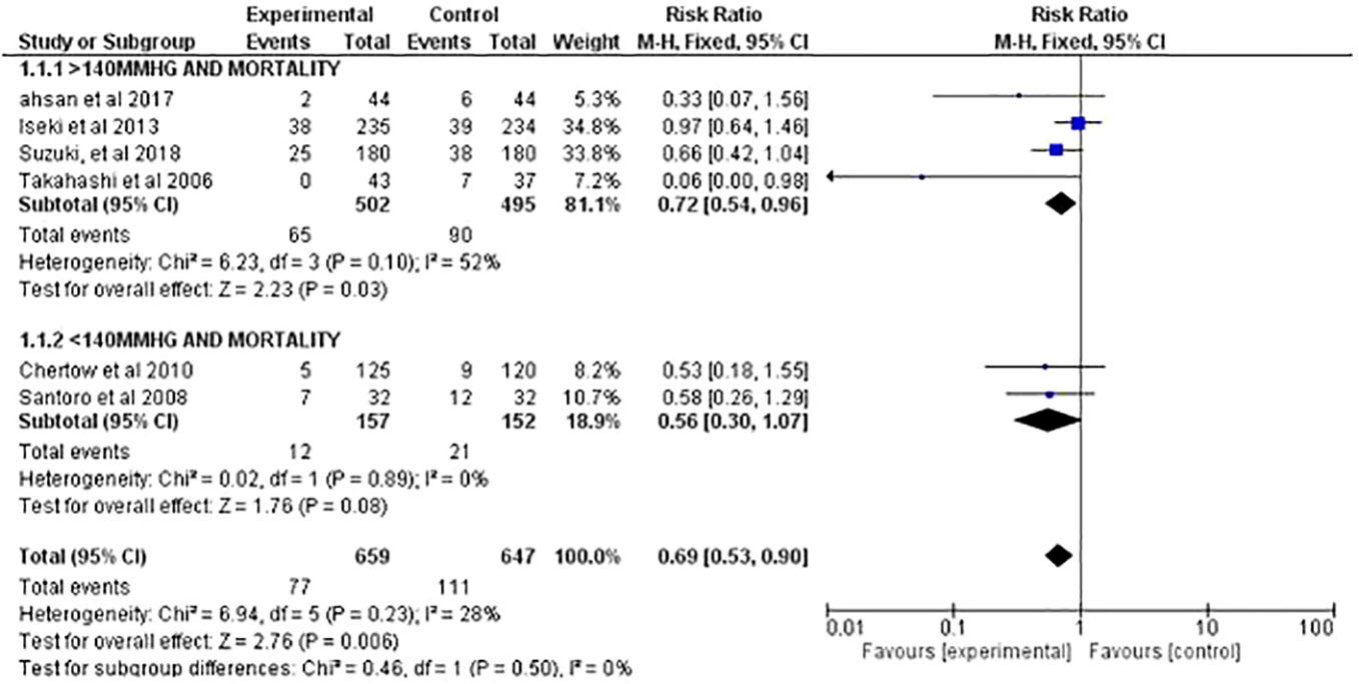
Quantitative assessment and sub-group analysis of selected studies.

The pooled RR for reduction in mortality of the two studies was 0.56 (95% CI, 0.3–1.07; *P* = 0.08). The results suggested that there was 44% of risk reduction in mortality rates among patients that were able to achieve systolic blood pressure <140 mm Hg. Overall, heterogenicity (I^2^, 0%) was negligible among the studies (**Figure 2**).

### Systolic Blood Pressure >140 mm Hg and Mortality

Four studies were able to achieve systolic blood pressure >140 mm Hg at the end of trial. All four studies employed pharmacological intervention in their trials. Altogether, there were 495 patients in control arm compared to 502 in the intervention arm. The total mortality among control arm patients was 90 (18.1%) compared to 65 (12.9%) in the intervention arm.

The pooled RR for reduction of mortality of the four studies was 0.72 (95% CI, 0.54–0.96; *P* = 0.003). The results suggested interventions applied to achieve systolic BP >140 mm Hg was able to significantly reduce the risk mortality by 28%. heterogenicity (I^2^, 52%) was moderate (**Figure 2**).

### Blood Pressure and Mortality

Overall, the pooled estimates of all the six included studies (with systolic BP range <140 and >140 mm Hg at the end of trial) suggested that the reduction in systolic blood pressure significantly reduce all-cause mortality (RR, 0.69%; 95% CI, 0.53–0.90, *P* = 0.006) among ESRD patients on hemodialysis. Thereby suggesting that the patients receiving any form of intervention to reduce blood pressure had 31% decreased risk of mortality compared to standard arm patients. Overall, heterogenicity (I^2^, 28%) was low, thereby suggesting there was not much variation among the selected studies (**Figure 2**).

### Biasness

**Figure 3:**
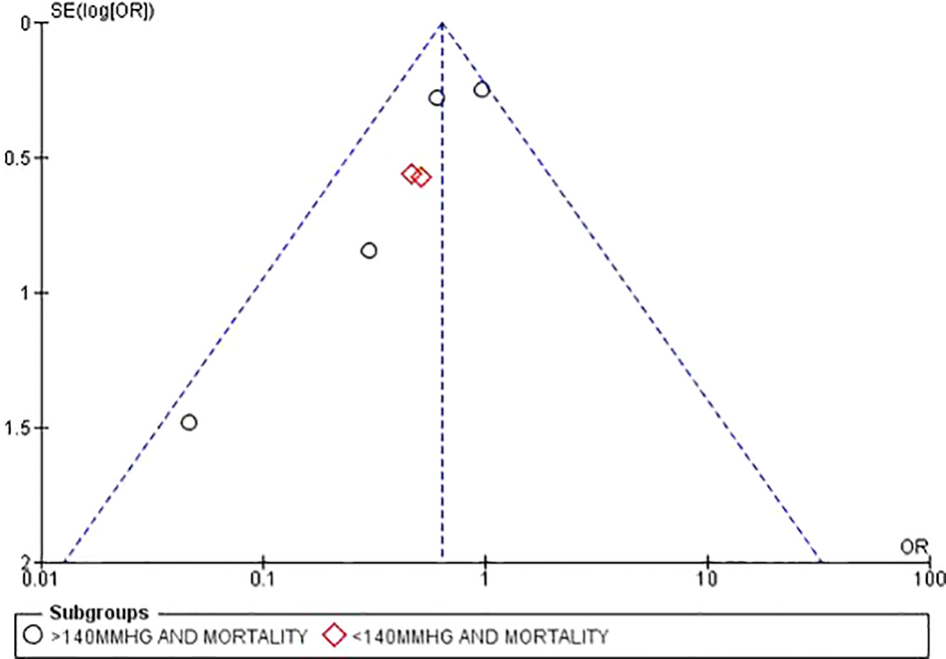
Study biasness based on funnel plot.

All included studies were well within the spread of the funnel plot thereby suggesting minimum biasness among the studies (**Figure 3**).

## Discussion

It is well established that uncontrolled high blood pressure is a powerful predictor of cardiovascular complications leading to mortality among the general population. However, the prevalence of cardiovascular complications leading to mortality among hemodialysis patients is even higher at 70% to 80% (Hannedouche et al., 2016). Clinical practice guidelines suggest a pre-dialysis blood pressure of <140/90 mm Hg and post-dialysis blood pressure of <130/80 mm Hg as targeted blood pressures for hemodialysis patients (K/DOQI Workgroup, 2005). However, there are some concerns regarding these targets, since most of the data is largely manipulated from observational studies from non-ESRD patients (Robinson et al., 2012). Hence, ideal blood pressures among hemodialysis patients remain unclear.

While most clinicians are aware that strict blood pressure control is necessary to achieve desired clinical outcomes in ESRD patient on hemodialysis, this is often not achieved. The main reason is blood pressure among ESRD patients on hemodialysis depends on multiple factors, such as patient's age and the presence of other comorbidities. Agarwal and Weir (2010) suggested that the rate of uncontrolled blood pressure among hypertensive hemodialysis patients is up to 70%, while 12% of hypertensive hemodialysis population is untreated, and 58% are inadequately treated (Hannedouche et al., 2016). At the same time, studies have indicated that strict blood pressure control in ESRD patients can lead to intradialytic hypotension and other adverse clinical outcomes especially among vulnerable group, such as old age, and those with multiple comorbidities (Iseki et al., 2013; Aftab et al., 2017). Given that blood pressure is one of the major factors in determining the prognosis and survival of patients on hemodialysis, this raises a question, what is the ideal blood pressure range among hemodialysis patients?

The current meta-analysis has taken into account an important parameter i.e. mortality, as an outcome of ideal blood pressure range among hemodialysis patients. As previous studies have identified systolic blood pressure is a powerful predictor of cardiovascular risk and mortality (Staessen et al., 1997; Gheorghiade et al., 2006; Boan Andrea et al., 2014), we have also focused on systolic blood pressure as a marker for mortality outcomes in our systematic review. Our analysis suggests that there was a 44% of risk reduction in mortality rates among patients that were able to achieve systolic blood pressure of <140 mm Hg. Meanwhile, patients that were able to achieve a blood pressure of >140 mm Hg, the mortality risk was significantly reduced by 28%. Importantly, our findings reflect that ideal blood pressure of <140 mm Hg is consistent with the National Kidney Foundation guideline recommendations. However, rather than extrapolating findings from observational studies involving non-ESRD patients, our findings have the advantage of providing a concrete evidence from RCTs involving ESRD patient on hemodialysis. Notably, the overall effect of our analysis suggests that there was a 31% decreased risk of mortality in hemodialysis patients with overall lowering of blood pressure thereby indicating the importance of blood pressure control among hemodialysis patients.

Another key factor identified in our analysis was that all studies that were able to reduce systolic blood pressure to <140 mm Hg employed non-pharmacological interventions while studies that achieved blood pressure target of >140 mm Hg employed pharmacological interventions. Among the non-pharmacological interventions, Santoro et al. (2008) studied the impact of n-line high-flux hemofilteration (HF) with ultrapure low-flux hemodialysis on survival among hemodialysis patients. The authors concluded that HF not only improved the survival among hemodialysis patients but also resulted in less hospitalization and less fluctuations in blood pressure control. These results are attributed by the authors mostly to B_2_ globulin removal, favorable sodium or negative thermal balance, removal of vasodepressor molecule or lower removal of vasoconstrictor molecule by the HF hemodialysis resulting in improved cardiovascular stability rather than direct lowering of blood pressure. Similarly, Chertow et al. (2010) studied conventional and frequent hemodialysis in improving patient outcomes. They concluded that more frequent hemodialysis sessions resulted in improved blood pressure management, decreased left ventricular mass, and improved survival. Both these studies demonstrated improved survival among hemodialysis patients that may not be directly related to blood pressure control but rather related to factors that improve cardiovascular stability that are closely related to ideal blood pressure. Therefore, ideal blood pressure attainment could not be neglected in both the cases.

On the other hand, studies that achieved blood pressure levels of >140 mm Hg employed Angiotensin Receptor Blocker (ARBs) as their pharmacological interventions. ARBs and Angiotensin-converting Enzyme Inhibitors (ACEIs) are both commonly used in clinical practice for ESRD patients as they are the first line antihypertensive agents recommended by the National Kidney Foundation KDOQI guidelines (Kidney Disease Outcomes Quality Initiative (K/DOQI), 2004). It is suggested that ARBs reduce left ventricular hypertrophy in hemodialysis patients and may be more potent than ACEIs (Shibasaki et al., 2002; Yang et al., 2013). An observational study reported that ARB in combination with another antihypertensive medication (but not an ACEI) may have a beneficial effect on cardiovascular mortality among ESRD patients on hemodialysis (Devolder et al., 2010). These may provide rationale for studies to employ ARB as their intervention to reduce blood pressure among ESRD patients.

The use of antihypertensive agents is one the prime strategies to manage blood pressure among hemodialysis patients. With reports also suggesting that antihypertensive agents may also be associated with low blood pressure and intradialytic complications, an unexpected point of use of these antihypertensive agents may well be paradoxically associated with higher blood pressure (Thomson et al., 1967; Agarwal and Weir, 2010). Excessive medications may well limit the opportunity of other blood pressure management strategies, such as achieving adequate dry weight and other non-pharmacological interventions and lead to resistant hypertension through expanded blood volume and other phenomenon respectively (Charra et al., 1996; Sinha and Agarwal, 2009). Thereby a combination of pharmacological and non-pharmacological interventions should be employed to achieve desired clinical outcomes in hemodialysis patients.

## Conclusion

The ultimate aim of renal replacement therapy is to improve patient survival and clinical outcomes. Surprisingly, there is no consensus on ideal blood pressure among ESRD patients on hemodialysis. Basis for this lack of consensus appears to be multifactorial that includes lack of agreement on best blood pressure measurement time related to the hemodynamic instability observed in this population. The current meta-analysis assessed survival as an outcome to establish the ideal blood pressure range among ESRD patients, thereby providing a rationale to use <140 mm Hg systolic blood pressure as an ideal target.

## Author Contributions

RA and RS have drafted the first version of the manuscript. All authors have been involved in all stages of the study design and have participated in writing the protocol whereas submission to PROSPERO was done by RS and RA. CM was involved in data extraction whereas RS and RA overlooked the process. Meta-analysis was performed by RA. IS was involved in qualitative analysis, manuscript write up, and critical review All authors approved the final manuscript. Data can be accessed by all the authors throughout and after the study.

## Funding

This study was funded by Taylor's University, Malaysia (TRGS/ERFS/2/2018/SOP/018). The source of funding had no role in the design, conduct or any influence in the study.

## Conflict of Interest Statement

The authors declare that the research was conducted in the absence of any commercial or financial relationships that could be construed as a potential conflict of interest.
